# Occurrence of pesticide residues and associated ecological risks assessment in water and sediment from selected dams in northern Ghana

**DOI:** 10.1371/journal.pone.0312273

**Published:** 2024-10-21

**Authors:** Abdou Orou-Seko, Dennis Chirawurah, Alexis Houndji, Felix Achana, Joyce Aputere Ndago, Matilda Nkansah-Baidoo, Martin Nyaaba Adokiya

**Affiliations:** 1 Department of Environmental and Occupational Health, University for Development Studies, Tamale, Ghana; 2 Research Laboratory in Aquaculture and Aquatic Ecotoxicology, University of Parakou, Parakou, Benin; 3 Department of Epidemiology, Biostatistics and Disease Control, University for Development Studies, Tamale, Ghana; 4 Department of Social and Behavioral Change, School of Public Health, University for Development Studies, Tamale, Ghana; 5 Department of Social and Behavioral Sciences, School of Public Health, University of Ghana, Legon, Ghana; 6 USAID Quality Services for Health Activity, Accra, Ghana; Fisheries College and Research Institute, INDIA

## Abstract

Pesticides are chemicals used to enhance crop production. However, their residues can persist and accumulate in various environmental settings. This study assessed the occurrence of pesticide residues and ecological risks in surface water and sediment from the Libga and Builpela dams in northern Ghana. A total of 42 pesticides were analysed. Toxic units were used to assess the acute risk to sediment-dwelling organisms and aquatic biota. Risk quotients were employed to assess the chronic risk to aquatic organisms. Chlorpyrifos, atrazine, profenofos, and chlorfenvinphos were the main chemicals found in surface water. The concentrations were highest near the Builpela dam (0.413μg/L, 2.927μg/L, 0.304μg/L, 0.692μg/L, 0.073μg/L, 0.290μg/L, 0.06μg/L, and absent in the Libga dam). In the sediment, the organophosphates pyrimiphos-methyl and chlorpyrifos were found at high concentrations. They were found in higher quantities in the Libga dam (0.554mg/kg and 0.052mg/kg, respectively) and Builpela dam (0.051mg/kg and 0.043mg/kg, respectively). For organochlorines, p,p’-DDE and p,p’-DDD were the main residues detected at high concentrations. However, concentrations were higher for p,p’-DDD at Builpela than for p,p’-DDE. Additionally, high concentrations of atrazine were detected in this study. The toxic units showed a high acute risk for organisms that live in sediment as a result of chlorpyrifosfos and chlorfenvinphos residues. Similarly, pirimiphos-methyl and chlorfenvinphos, followed by chlorpyrifos, contributed to high acute risk in aquatic invertebrates. Risk quotients showed that both dams had a high long-term risk for aquatic life; however, the risk was higher at the Builpela dam due to Pirimiphos-methyl and Chlorfenvinphos. Ghana’s pesticide regulations are less comprehensive and enforcement is often weaker in protecting aquatic organisms. It is recommended to enforce strict limits on pesticide residues. Additionally, there is a need to regularly review and update these regulations based on new scientific data to protect aquatic ecosystems.

## Introduction

Globally, pesticide residues are a threat to one health [1]. Following their application in agricultural areas, pesticides are distributed and conveyed to different locations via mechanisms such as spray drift, groundwater leaching, wind, and direct runoff [2], resulting in pollution. The extent of this drift varies based on weather conditions, applicator machinery, and the type of chemical applied. Despite technological advancements and the goal of risk reduction, drift remains uncontrollable. This makes contaminants ubiquitous worldwide, including in Antarctica [3]. Thus, pesticides are prevalent in the environment and have a significant influence on biodiversity, posing a hazard to aquatic systems worldwide [4]. Pesticide contamination is linked to an increase in pesticide usage and intensified agricultural production systems. In 2021, the European Union sold 355,175 metric tons of pesticides. This is an increase of 2.7% compared to the previous year’s sales of 346,000 metric tons. The agricultural sector accounts for the majority of these sales [5]. The Russian Federation is the primary consumer, with Spain, France, Italy, and Germany following suit [6]. However, the Food and Agriculture Organization (FAO) estimates that 4.12 million metric tons of pesticides are used annually worldwide. Africa accounts for 2% of this amount [7]. In contrast to other regions, 30% of the pesticides used in Africa are insecticides. The main pesticide users in Africa are Cameroon, South Africa, and Ghana [8].

In Ghana, the agricultural land area spans over 13.6 million hectares (57%) [9]. This makes agriculture the largest land use type in the country. Out of this, about 6.8 million hectares (50%) are used for agriculture, while 222,978 hectares are irrigated [9]. Pesticides which are regularly employed for plant protection, are recognised as indispensable for enhancing both the quality and quantity of agricultural harvests [10]. The main agricultural commodities that are frequently treated with pesticides include oil palm, cotton, coffee, cocoa, and vegetables [11]. According to Doris [12], over 9,600 metric tons of pesticides were applied in Ghana in 2021. The extensive application of pesticides in agricultural environments may have harmful effects on both aquatic life and human populations in close proximity [13]. Upon entering water bodies, these compounds can exhibit toxicity towards aquatic organisms. Over time, they can also modify the composition of invertebrate and fish communities. This leads to a decline among more vulnerable species and an increase among those that are more resistant. Additionally, these compounds can impede the growth of algae and result in reduced biodiversity [14, 15]. Generally, sediment level is the preferred food source for aquatic biodiversity. It acts as a trap for the most dangerous contaminants that can contaminate and accumulate in the tissues of aquatic organisms. Furthermore, the level of fish contamination is influenced by the degree of pollution in the water. Consequently, fish serves as the main bioindicator for detecting pesticide pollution in water bodies [16, 17]. Thus, the excessive accumulation of toxins in fish might serve as an exposure and health risk to consumers.

The significant mobility of pesticides in aquatic media leads to environmental concerns that have health implications. It is crucial to conduct monitoring studies to determine the distribution of these chemical compounds in the environment [18], and assess their ecological and health risks. While the majority of chemicals released in the vicinity of surface waters ultimately accumulate in the sediment, there is limited literature on the occurrence of pesticides and their potential ecological risks in this particular setting. Few studies have been conducted locally which encompassed the simultaneous detection of several current-use and banned pesticides in different matrices of the aquatic environment. Additionally, few previous studies used the data to assess the potential risk to biota in agricultural, urban, and industrial land use. Thus, the objectives of this study were: 1) to evaluate the occurrence of 42 pesticides used in agriculture at different environmental matrices (sediments and surface water) of two important dams, and 2) to assess the chronic and acute toxicity risk for sediments and aquatic organisms using the Toxicity Unit (TU) and Risk Quotient (RQ), respectively, of two selected dams in northern Ghana.

## Materials and methods

### Study area

**Fig 1:**
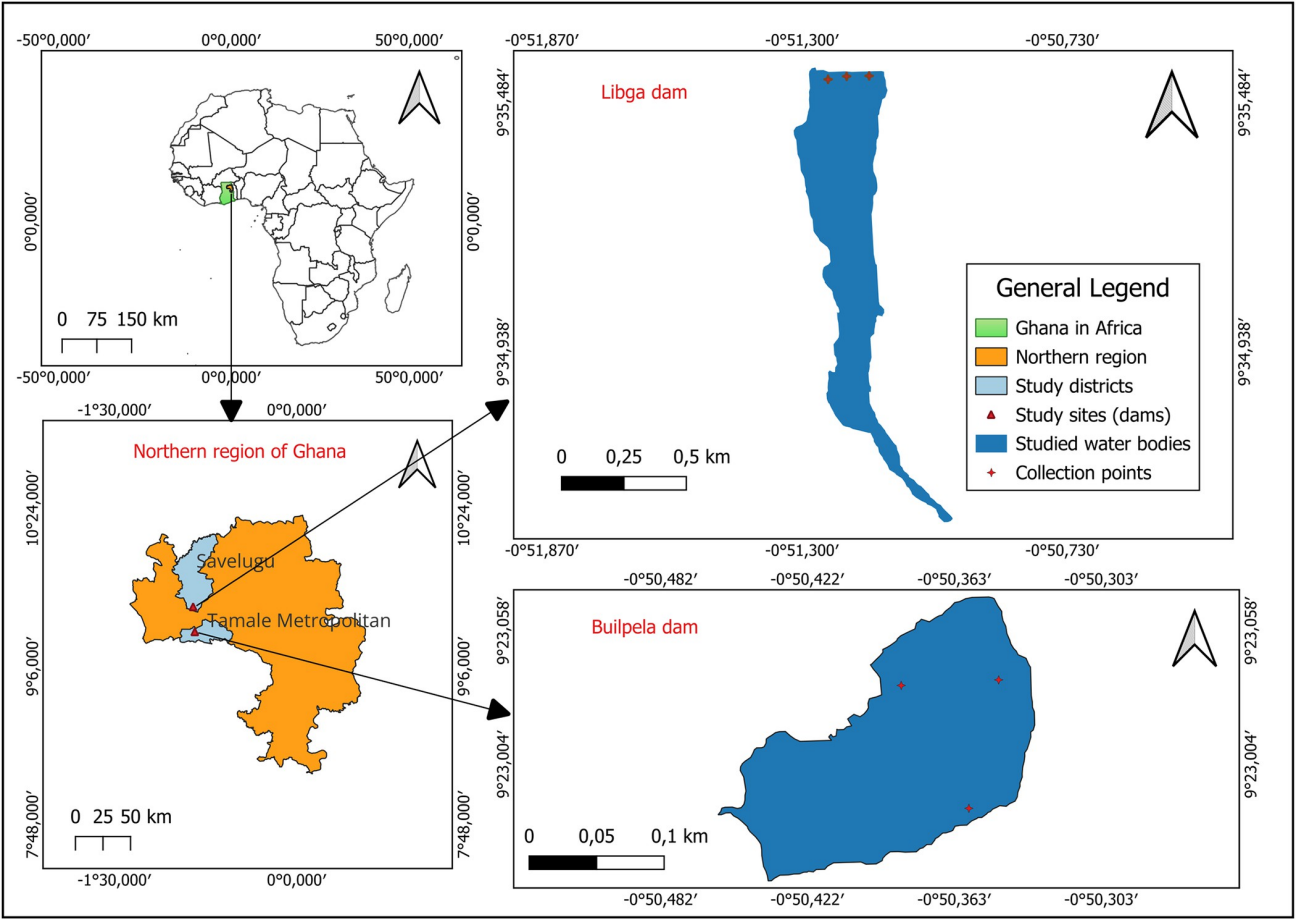
Study map showing Libga and Builpela dams in the northern region of Ghana.

The northern region of Ghana has a monomodal rainfall pattern, starting in May and ending in October, with an annual rainfall range of 900–1000 mm [19]. The main crops cultivated in the region include maize, rice, millet, sorghum, cassava, yam, peanuts, cowpeas, and soybean [20]. The most widely used pesticides in the area are herbicides, insecticides and fungicides. These pesticides are under the supervision and control of irrigation authorities [21]. In this area, approximately 80% of the employment opportunities are connected to agriculture using conventional agricultural methods. This makes agriculture the main industry in the region [22]. The study data were collected from the Libga dam in Savelugu Municipality and Builpela dam in Tamale Metropolis of the northern region of Ghana (Fig 1). The dam characteristics are presented in Table 1. Libga is a medium-sized dam with a maximum area of 48 hectares [23] and it is located between 9° 35’ 20.8818"N and 0° 51’ 13.6398"W. Builpela is a small dam and it is located between 9° 23’ 0.4374"N and 0° 50’ 23.1756"W. These dams are located along the banks of the White Volta subbasin in northern Ghana. The dams in this study were selected based on the following criteria:

- Inhabiting biodiversity: Compared with the Bontanga dam, the largest dam in the area, the Libga dam showed higher values for all fish species diversity indicators. Among these metrics are species evenness (0.52 vs. 0.40), species diversity (2.4 vs. 1.6), and species richness (2.4 vs. 1.1) [24].
- These dams have not been desilted since their construction.
- Economic importance: The selected dams currently sustain a local fishing industry that supplies seafood for local community consumption.
- The close proximity of farm fields to dams may lead to water contamination because of pesticide use.
- Water supply: The Libga dam receives water from the White Volta River, which passes through most Ghana’s agricultural villages during periods of flooding and precipitation. Precipitation that travels through nearby homes and agricultural areas (vegetable farms) replenishes the Builpela Dam.

**Table 1:** Characterization of the sampling sites.

Sampling site (Community type)	Land use	Main purpose of the dam	Distance between site (km)
Libga dam (Rural)	• Agricultural with crop in upstream such as cassava, soja, millet, groundnuts, sorghum and maize and downstream such as onions, tomatoes, pepper, amaranth, and rice production • Livestock grazing on natural pasture around the dam	Agricultural and irrigation	0
Builpela dam (Urban)	• Residential and recreational • Urban horticulture: crops such as tomatoes, peppers, amaranth etc. • Industrial	Agricultural (previously for community water supply)	24 (to site 1)

### Sample collection

The sample was collected from November 16, 2023 to December 11, 2023. Surface water samples were collected from different locations within each dam at a depth of approximately 1m (depth at which the sample collector stood, measured from the embankment) into previously cleaned 1L plastic bottles and covered with screwed caps. Three water samples were collected from each of the dam. Each sample consisted of three subsamples, each measuring 335ml, collected from three different points in the dam. The sampling points were spaced 50 feet apart. The three subsamples were combined to form a 1L pooled sample. The subsamples were obtained by immersing the sampling bottles at a depth of 0.2m below the water surface, ensuring that they were completely filled without any air space in the bottleneck.

Sediment samples were collected from the bottom of the dam using a stainless-steel scoop at the same locations of1m depth from the embankment. A triplicate sample weighing 1kg was collected from each dam, starting downstream and moving upstream. Each sample comprised three identical 335g samples collected at three distinct sampling points inside the dam. Subsequently, all grab samples were placed in a thoroughly cleaned plastic basin and meticulously homogenised. Prior to placement in the collection basin, stones, roots, debris, foreign objects, and shells were eliminated from the sample. Water was poured from each sample grab. To obtain the final sample, 1kg of the mixture was weighed using a Fishfun 110 lb/50kg electronic scale. The measured amount was placed in a clean polyethylene bag.

Upon completion of the collection process, the samples (water and sediment) were wrapped in aluminium foil, appropriately labelled, sealed, and stored in a thermo-insulated container with ice blocks at 4 ± 2°C in the field. They were then transported to the pesticide residue laboratory of the Ghana Standard Authority and stored at −20°C until processing and analysis.

In this study, no permit was required to access the field sites. The study was conducted in public areas where no specific permissions are mandated for research activities.

### Pesticide occurrence assessment

Pesticide residue analysis was performed using the method described by Akoto et al. [25], as follow:

#### Sample preparation

The water samples were filtrated using 0.45μm fibre glass filters (Whatman) to eliminate any suspended contaminants. A 250μm stainless steel mechanical shaker was used to filter the sediment samples after they were air-dried.

#### Extraction of pesticide residues in water

Approximately 50mL of n-hexane was added to a separating funnel of 1L containing a filtered water sample of approximately 100mL. The solution was vigorously agitated for 5 minutes and then allowed to settle. Once the separation was complete, the organic phase was transferred into a 250mL conical flask, and the aqueous phase was extracted twice with 50mL of n-hexane. The organic phase obtained was mixed and dehydrated by passing it through a glass funnel filled with activated anhydrous sodium sulphate. The organic portion was subsequently condensed to a nearly dry state by using a vacuum rotary evaporator at 40°C.

#### Pesticide residues extraction in sediment

A dried sediment sample weighing 10g was introduced into a cleaned extraction thimble, along with acetone and n-hexane. The mixture was then dehydrated in an oven. The extraction of pesticide residues from sediments was performed using a combination of 150mL of n-hexane and acetone at a ratio of 4:1 v/v for 6 hours using the Soxhlet extraction method. The extract was subsequently condensed to a nearly dry state by using a vacuum rotary evaporator at 40°C. Subsequently, using a Florisil® cartridge (1 g, 6 mL, 60–100 μm particle size, 60 Å pore size) each extract was diluted in 10mL n-hexane and cleaned.

#### Sample clean-up

Anhydrous sodium sulphate (1.0g) was added to a glass column (approximately 10 cm in length and 0.5 cm in internal diameter) with activated silica gel (2.5g) and sealed with glass wool. A volume of 10mL n-hexane was used to moisten and clean the column. The sample was subsequently placed on a column and separated using 20mL of a hexane/acetone mixture. The recovered eluates were transferred to a flask with a circular bottom and subsequently evaporated until completely dry. Next, the residues were dissolved in 2mL of ethyl acetate and transferred to a gas chromatography vial for analysis.

#### Gas chromatograph analysis

A Varian CP-3800 gas chromatograph (Varian Association Inc. USA) with an 63Ni Electron Capture Detector was used to analyse the level of residues of organochlorine pesticides (OCP). The separation was performed using an SGE BPX-5 capillary column with a length of 60m, internal diameter of 0.25mm, and film thickness of 0.25μm. The column was equipped with a 1m retention gap. The oven temperature was set according to the following instructions. The initial temperature was set to 90°C and held for 3 minutes. The temperature was then increased at a rate of 30°C/min to reach 200°C and maintained for 15 minutes. Subsequently, the temperature was increased to 265°C at the rate of 5°C/minutes for 5 minutes. Finally, the temperature was increased to 275°C at a rate of 3°C/min and left to stabilise for 15 minutes. The injector was configured in pulsed split less mode operating at a temperature of 250°C and a pressure of 1.441 bar. The pulsed pressure applied was 4.5 bar for 1.5 minutes. The purge flow rate was 55.4mL/minutes for 1.4 minutes. The temperature of the detector was set at 300°C. Nitrogen was used as the carrier gas at a flow rate of 30mL/minutes.

The levels of organophosphate pesticide residues (OP) were measured using a Varian CP-3800 GC integrated with a Combi PAL Auto sampler. The column employed was a fused silica capillary with a diameter of 0.25mm and a length of 30m. It was coated with VF-1701, a film with a thickness of 0.25μm. The oven temperature was set up as follows: it started at 70°C and rose steadily at 25°C per minute to 200°C after 6 min. Subsequently, the temperature was increased from 20°C/minutes to 250°C and maintained at that level for 19 minutes. The injector was configured to operate in the pulsed splitless mode at 270°C. The detector was operated at a consistent temperature of 280°C in "constant makeup flow" mode. Nitrogen gas was employed as the carrier gas and flowed at a rate of 2mL/minutes.

#### Quality assurance and control

Examining solvent and procedure blanks helped implement quality control procedures. All reagents used were of analytical grade. The samples were examined in triplicate and the mean concentrations were estimated based on the number that yielded positive results. These were then used to confirm the precision of the method. Recalibration curves were obtained for every batch of samples to confirm their correlation coefficients and consistently maintained at a minimum value of 0.98. The limits of quantification (LOQ) for pesticides were determined to be 0.01 mg/kg for sediment and 0.05 μg/L for water in the study. The assurance analysis results confirmed that the pesticide determinations were accurate and within allowed values.

### Ecological risks assessment

#### Short-term (acute) risk toxicity assessment

To determine the acute risk to sediments and surface water, the physicochemical and toxicity properties of pesticides were acquired from the Pesticide Properties Database [26] and the United States Environmental Protection Agency ECOTOX database [27] (S1–S3 Tables). For each pesticide evaluated, we extracted physicochemical properties such as linear sorption coefficient (Kd) when available, the organic carbon-water partitioning coefficient (Koc). The acute and chronic toxicity data extracted, included the LC50 (median lethal concentration) and EC50 (median effective concentration) values for the relevant aquatic and sediment-dwelling organisms. The ecological risk assessment was conducted for acute risk in aquatic and sediment-dwelling organisms. This was based on the data collected from literature on acute and chronic toxicity in reference organisms and the pesticides analysed.

Toxic units (TUs) were computed to assess the individual impact of each pesticide on the immediate risk of toxicity to organisms residing in the sediment. TUs were determined based on the equation [28]:

$$TU=\frac{Ci}{L(E)C50i}$$

Ci is the concentration of pesticide (i) in the sample, and L(E)C50i represents the median lethal or effective concentration of pesticide (i) in aquatic or sediment-dwelling organisms.

To assess the overall toxicity of pesticides at each sampling site, the total toxic units (ΣTU) were calculated using the following equation [29].

$$\sum TUsite=\sum_{i=1}^{n}TUi$$

where TUi represents the Toxic Unit of pesticide i at the study site. A ΣTU value greater than one signifies the presence of ecological danger at the station.

To assess the acute risk in sediment, the calculations were based on the 96-hour LC50 values for *Chironomus* (C) *riparius*. This is a species that resides in sediment. In cases where there were no toxicity data available for pesticides for *C*. *riparius*, the 48-hour EC50 values in *Daphnia (D) magna* were utilised (cases of Profenofos, Chlorfenvinphos, Aldrin, Heptachlor, Dieldrin, and p,p′-DDD). Despite not being classified as a sediment-dwelling organism, *D*. *magna* has been utilised in sediment toxicity research because of its exceptional sensitivity and direct correspondence with LC50 values in *C*. *riparius* [28–30]. In the absence of toxicity data for *C*. *riparius* and *D*. *magna*, the LC50 or EC50 values were assigned the same value as those of the parent compounds [28]. This was the case for β-HCH, for which the LC50 value for *Chironomus riparius* in Lindane was used.

To determine TUs, the pesticide concentrations in the sediment were transformed into pore water concentrations (Cpw) using the equation provided by [29]:

$$Cpw=\frac{Cs}{Kd}$$

Cs represents the concentration of pesticides detected in the sediments and Kd is the linear sorption coefficient. To determine the Kd value, the fraction of organic carbon (foc) and the organic carbon-water partitioning coefficient (Koc) in the sediments were calculated as follows:

Kd=Koc×foc

Koc was determined by utilising Kow, which represents the octanol-water partition coefficient of each pesticide, as per the following equation:

logKoc=0.72(logKow)+0.49

To assess the acute risk in surface water, we used short-term endpoints for three different levels of the food chain: fish, invertebrates, and algae. Specifically, we calculated the cumulative toxic units (∑TU) for fish, invertebrates, and algae. For fish, we employed the 96-hour LC50 values in *Oncorhynchus mykiss*. For aquatic invertebrates, we employed the 48-hour EC50 values obtained from immobilisation tests conducted with *Daphnia magna*, except for p,p’-DDE, where we used the value from *Bosmina longirostris*. Lastly, for algae, we relied on EC50 values derived from 72-hour growth inhibition tests.

#### Aquatic risk (long-term effects) assessment

Evaluation of the chronic risk to sediment-dwelling organisms was not possible due to lack of toxicity data. To determine the chronic aquatic risk in surface water, the RQ equation was used as outlined in the Technical Guidance Document on Risk Assessment from European guidelines [31]. This allowed to evaluate the potential long-term pesticide risks in water for three representative taxa in the aquatic ecosystem: fish, invertebrates, and algae.


$$RQ=\frac{MEC}{PNEC}$$

The variables MEC and PNEC represent the measured environmental concentration and predicted no-effect concentration for each pesticide, respectively. The Predicted No-Effect Concentration (PNEC) was calculated using the following equation.


$$PNEC=\frac{CC}{AF}$$

The critical concentration (CC) is the lowest observed value of the no observable effect concentration (NOEC) among the analysed trophic levels. AF refers to the assessment factor. The AF is used as an evaluation criterion to measure the degree of uncertainty in extrapolating toxicity estimations obtained from laboratory tests (conducted on a defined number of species) to actual environmental circumstances in the real world [29].

In cases where there were no NOECs for any of these taxa, the lowest value of L(E)C50 from the PPDB [26] was used [32]. The method suggested by Papadakis et al. [38] was used to set the AFs for aquatic organisms. In this study, 10 AFs were used when three NOECs were available, 50 AFs when two NOECs were available, 100 AFs when only one NOEC value was available (for fish or aquatic invertebrates), and 1000 AFs when there was no available NOEC value and an LC50 or EC50 was used. This was done to account for the uncertainty associated with accuracy, model errors, and lack of toxicity data [33].

Ultimately, the concentration addition effect model was employed to classify various sample sites based on long-term risk accumulation. The summation of the RQ (ΣRQ) was computed using the equation provided by Pérez et al. [28].


$$\sum RQsite=\sum_{i=1}^{n}RQi$$

The risk quotient for pesticide i in the analysed station is denoted as RQi.

When the sum of the risk quotient (RQ) is less than one, the likelihood of chronic toxicity at the assessed location is minimal. Values for ΣRQ between 1 and 10 indicate a medium level of risk, whereas values above 10 indicate a high level of risk [34].

### Statistical analysis

Descriptive statistics were performed using SPSS version 26.0 (SPSS Inc. 2008). The results were presented as mean level of pesticides detected at each site and range. The individual acute and chronic risks of pesticides, the overall risk (toxic units and risk quotient) associated with each sampling site, and the contribution of specific pesticides to the acute and chronic risks were calculated, and figures were generated using Excel 2016.

#### Ethical consideration

The study received ethical approval from the Committee of Human Research and Publication Ethics (CHRPE) of the Kwame Nkrumah University of Science and Technology (KNUST) Ref: CHRPE/AP/864/23. This study does not involve data collection from human participants and thus, obtaining informed consent was not required.

## Results

### Pesticide occurrence

#### Pesticide occurrence in surface water

In this study, we analysed 42 pesticides. These include 14 organochlorine, 8 organophosphate, and 20 others currently used pesticides in surface water samples from the Libga and Builpela dams in Northern Ghana. The detection frequency (DF) and mean concentration levels (range) are presented in Table 2. Among the 5 organophosphates analysed, 5 pesticides were detected (Profenofos, Chlorfenvinphos, Chlorpyriphos, Diazinon, and Pirimiphos-methyl). Chlorpyriphos showed the highest detection frequency (DF) at 100% in both locations, with mean concentrations higher in the Libga dam (0.07μg/L, range 0.07–0.08μg/L) than in the Builpela dam (0.41μg/L, range 0.13–0.90μg/L). These levels exceed the EU guideline limit of 0.1μg/L. Profenofos was detected in 33% of samples from Libga dam and in 100% of samples from Builpela dam, with a mean level of 0.30μg/L (range 0.05–0.80μg/L) lower than that of Libga dam (0.06μg/L). This was followed by chlorfenvinphos (in terms of concentration). This chemical was not detected in the Libga dam but was found in 33% of the Builpela dam samples at 0.69μg/L. Diazinon and pirimiphos-methyl were detected less frequently and in lower concentrations. Additionally, out of the 14 organochlorine pesticides analysed only, p,p’-DDE was found in 33% of Libga dam samples and in 67% of Builpela dam samples, with a mean level higher in Builpela (0.07μg/L, range 0.06–0.08μg/L) than in Libga (0.05μg/L). Atrazine, a triazine herbicide, was detected in all samples from both locations, with a mean concentration in Builpela higher than that of Libga (2.93μg/L, range 2.18–4.02μg/L and 0.29, range 0.08–0.50μg/L, respectively).

**Table 2:** Summary of pesticide residue concentrations in surface water samples from the Libga and Builpela dams in northern Ghana.

Compound	Water μg/L				
	Libga dam		Builpela dam		
	DF (%)	Mean level (range) of sample above LOQ	DF (%)	Mean level (range) of sample above LOQ	EU^a^ Guideline limit
**Organophosphate**					
Profenofos	33	0.06 (0.060)	100	0.30 (0.05–0.80)	0.1
Chlorfenvinphos	n.d	n.c	33	0.69 (0.69)	0.1
Chlorpyriphos	100	0.07 (0.07–0.08)	100	0.41 (0.13–0.90)	0.1
Diazinon	67	0.05 (0.05–0.06)	33	0.06 (0.06)	0.1
Pirimiphos-methyl	33	0.05 (0.05)	33	0.07 (0.07)	0.1
**Organochlorines**					
p,p’-DDE	33	0.05 (0.05)	67	0.07 (0.06–0.08)	0.1
**Triazine**					
Atrazine	100	0.29 (0.08–0.50)	100	2.92 (2.18–4.02)	2

^a^ EU directive 2013/39/EC (Water Quality Limit), LOQ: Limit Of Quantification, n.d: not detected, n.c: not calculated, DF: detection frequency

#### Pesticide occurrence in sediment

This study investigated the concentrations of pesticide residues in sediments from the Libga and Builpela dams in northern Ghana (Table 3). Among the 8 organophosphate pesticides analysed in the sediment samples, 4 were detected (profenofos, chlorfenvinphos, chlorpyriphos, and pirimiphos-methyl) at both sites. Pirimiphos-methyl showed the highest detection frequency at 100% in both locations, with the mean concentration being higher in Libga (0.55mg/kg, range 0.03–0.85mg/kg) than in Builpela (0.05mg/kg, range 0.02–0.07mg/kg). Profenofos was detected in 67% of samples from both dams. Chlorfenvinphos was detected in 67% of samples from the Libga dam and in 67% and 100% of samples from the Builpela dam. However, Profenofos showed a higher concentration in Builpela (0.07mg/kg, range 0.03–0.10mg/kg) than in Libga (0.04mg/kg, range 0.02–0.06mg/kg). Chlorfenvinphos showed similar mean concentrations at both sites (0.04mg/kg, range 0.04–0.05mg/kg in Libga and 0.04mg/kg, range 0.13–0.98mg/kg in Builpela). This was followed by chlorpyriphos, which was less frequently detected but showed a higher concentration in Libga (33%, 0.05mg/kg, range 0.05mg/kg) than in Builpela (100%, 0.04mg/kg, range 0.13–0.98mg/kg). Among the organochlorine pesticides investigated, 6 out of 14 were detected (aldrin, β-HCH, Heptachlor, Dieldrin, p,p’-DDE, and p,p′-DDD). Among them, p,p′-DDD was consistently detected (100%) in all samples from both locations. The concentration in Builpela dam was 0.62 times higher than in Libga’s level (1.37mg/kg, range 1.03–1.7mg/kg and 0.84mg/kg, range 0.57–1.04mg/kg, respectively). Subsequently, Heptachlor and p,p’-DDE were detected at high frequencies (67% in Libga and 100% in Builpela) at generally higher levels in Builpela dam. Dieldrin was only detected in Builpela dam at 0.71 mg/kg in 33% of the samples. Atrazine was consistently detected in 100% of samples from both locations. However, the concentration in Builpela dam was 0.44 times higher than in Libga dam (1.08mg/kg, range 1.04–1.11mg/kg, and 0.74mg/kg, range 0.50–1.13mg/kg, respectively).

**Table 3:** Summary of pesticide residue concentrations in sediment samples from the Libga and Builpela dams in northern Ghana.

Compound	Sediment (mg kg-1)						EU guideline limit (μg/L)^b^
	Libga dam			Builpela dam			
	DF (%)	Mean level (range)	Cpw (μg/L)^a^	DF (%)	Mean level (range)	Cpw (μg/L)^a^	
**Organophosphate**							
Profenofos	67	0.04 (0.02–0.06)	1.2	67	0.07 (0.03–0.10)	1.82	0.1
Chlorfenvinphos	67	0.04 (0.04–0.05)	3.55	100	0.04 (0.03–0.06)	3.43	0.1
Chlorpyriphos	33	0.05 (0.05)	0.41	100	0.04 (0.13–0.98)	0.34	0.1
Pirimiphos-methyl	100	0.55 (0.03–0.85)	29.63	100	0.05 (0.02–0.07)	2.58	0.1
**Organochlorines**							
Aldrin	33	0.06 (0.062)	0.21	100	0.07 (0.06–0.08)	0.23	0.03
β-HCH	100	0.28 (0.07–0.68)	12.78	67	0.35 (0.08–0.61)	15.09	0.1
Heptachlor	67	0.34 (0.12–0.56)	0.830	100	0.67 (0.05–1.07)	1.55	0.03
Dieldrin	n.d	n.c	n.d	33	0.71 (0.71)	3.29	0.03
p,p’-DDE	67	0.85 (0.79–0.91)	0.02	100	0.78 (0.1–1.21)	0.02	0.1
p,p′-DDD	100	0.84 (0.57–1.04)	0.38	67	1.37 (1.03–1.71)	0.58	0.1
**Triazine**							
Atrazine	100	0.74 (0.50–1.13)	437.06	100	1.08 (1.04–1.11)	598.33	2

^a^ Cwp: concentration in pore-water based on the mean value calculated in sediment

^b^ EU directive 2013/39/EC limit for water quality, n.d: not detected, n.c: not calculated, DF: detection frequency

Prior to calculating the acute risk in the sediment, it was necessary to convert the pesticide concentration into pore water. These results are shown in Table 3. The results showed that among the organophosphates, profenofos was present at 1.2 μg/L in Libga dam and 1.82 μg/L in Builpela dam, while chlorfenvinphos displayed similar concentrations in both dams (3.55 μg/L and 3.43 μg/L, respectively). Chlorpyriphos was detected at relatively lower levels (0.41 μg/L in Libga and 0.34 μg/L in Builpela), whereas pirimiphos-methyl showed a marked difference between the two sites, with 29.63 μg/L in Libga and 2.58 μg/L in Builpela. Among the organochlorines, aldrin was detected at 0.21 μg/L in Libga and 0.23 μg/L in Builpela. β-HCH levels were notably high in both dams, with concentrations of 12.78 μg/L and 15.09 μg/L, respectively for Libga dam and builpela dam. Heptachlor concentrations were also higher in Builpela (1.55 μg/L) compared to Libga (0.83 μg/L). Dieldrin was not detected in Libga but was present at 3.29 μg/L in Builpela. Both p,p′-DDE and p,p′-DDD were found in similar concentrations at both sites, with p,p′-DDE detected at 0.02 μg/L and p,p′-DDD slightly higher in Builpela (0.58 μg/L) than in Libga (0.38 μg/L). Lastly, atrazine showed remarkably higher concentrations than all other compounds, with 437.06 μg/L in Libga Dam and 598.33 μg/L in Builpela Dam, indicating significant contamination levels at both sites.

### Ecological risk assessment

#### Acute risk assessment

The toxic unit values for the sediment-dwelling organisms and aquatic biota at each site are listed in Table 4. The findings indicated that the ∑TU for sediment-dwelling organisms was greater in the Libga dam (32.88) than in the Builpela dam (29.04). The ∑TU values at both sites exceeded 1, indicating significant ecological danger at both locations. The total toxicity units (∑TU) for fish at both sites were lower than one (0.01 and 0.02, respectively) for the Libga and Builpela dams. These results indicate that there was no significant risk of acute toxicity for fish at either location. Similarly, the ∑TU for algae was higher in Builpela dam (0.05) than in Libga dam (0.01). Though both values remained below the threshold, indicating no acute toxicity risk to algae. In contrast, the ∑TU for invertebrates was significantly greater than 1 at both sites, with a higher ∑TU in the Builpela dam (7.38) than in the Libga dam (1.09). These findings suggest that invertebrates face a significant ecological risk at both locations, with the risk being particularly elevated in Builpela dam.

**Table 4:** Toxic units for sediment-dwelling organisms and aquatic biota from the Libga and Builpela dams in northern Ghana.

Sites	∑TU^a^ for sediment organisms	∑TU^a^ for aquatic biota			∑RQ^b^ for aquatic biota
		Fish	Invertebrate	Algae	
Libga dam	32.88	0.01	1.09	0.01	43.88
Builpela dam	29.04	0.02	7.38	0.05	163.12

^a^ ΣTU< 1 means no acute toxicity, and ΣTU> 1 means there is an acute toxicity.

^b^ When ΣRQ< 1, indicates chronic toxicity was minimal, Values of 1≤ΣRQ≤10 indicate medium risk, and ΣRQ>10 there is high risk.

#### Chromic risk assessment

The chronic toxicity risk assessment of pesticide residues in surface water for aquatic biota at Libga and Builpela dams through the risk quotients (∑RQ) is shown in Table 4. The results indicated a ∑RQ of 43.88 for Libga dam and a significantly higher ∑RQ of 163.12 for Builpela dam. According to the risk assessment criteria, both sites exhibited a high risk of chronic toxicity to the aquatic biota (10<**∑**RQ**)**. However, the risk is significantly higher at Builpela dam, with a ∑RQ approximately four times greater than that of the Libga dam.

#### Pesticide contribution to acute and chronic risks

**Fig 2:**
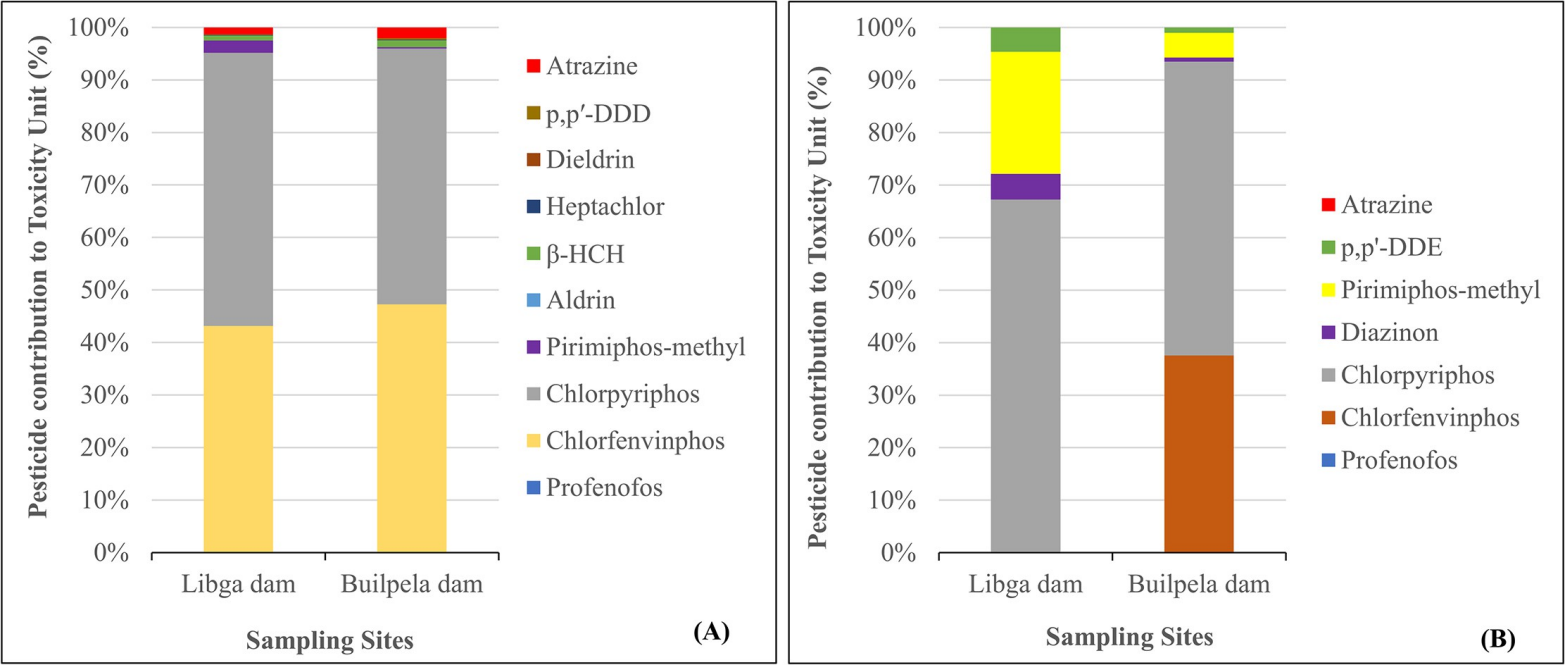
Pesticide contributions to toxic units in sediment (A) and surface water (B) from Libga and Builpela dams in northern Ghana.

The contribution of each pesticide to the ∑TU corresponding to each site in the sediment is shown in Fig 2(A), and for water in Fig 2(B). As shown in Fig 2(A), chlorpyriphos emerged as the dominant contributor to ∑TU in sediment at both locations, accounting for 52.04% in the Libga dam and 48.72% in the Builpela dam. Chlorfenvinphos was the second most significant contributor, with 43.14% in the Libga dam and 47.25% in the Builpela dam. The combined impact of chlorpyriphos and chlorfenvinphos approached almost 96% of ∑TU at both sites. Atrazine contributed more to ∑TU at the Builpela dam (2.06%) than at the Libga dam (1.33%). The contributions of Profenofos, Aldrin, β-HCH, Heptachlor, and Dieldrin were relatively minor, constituting less than 2% at both sites. The pesticide p,p’-DDE did not contribute to the ∑TU at any site, whereas p,p′-DDD had a minimal impact, with contributions of 0.13% in the Libga dam and 0.22% in the Builpela dam.

Fig 2(B), shows how different pesticides affected the number of toxic units in surface water for invertebrates. Chlorpyriphos had the biggest effect, making up 67.25% of TU at the Libga dam and 55.99% at the Builpela dam. Chlorfenvinphos were in second, making up 37.53% of TU at the Builpela dam but not at the Libga dam. Pirimiphos-methyl was the third-largest contributor in the Libga dam (23.25%). However, its contribution was lower in Builpela dam (4.71%). Chlorpyriphos and Pirimiphos-methyl alone contributed almost 90% to the ∑TU in the Libga dam and 60% to the ∑TU Builpela dam. Similarly, the three largest contributors (Chlorpyriphos, Chlorfenvinphos, and Pirimiphos-methyl) at Builpela dam were 98.23%. Diazinon and p,p’-DDE had a minor impact on the total toxic unit (∑TU) at both sites, contributing 4.88% to the ∑TU in the Libga dam and 0.77% in the Builpela dam. Similarly, they contributed 4.61% to the Libga dam and 0.99% to the Builpela dam. Profenofos and atrazine had a negligible impact on the total toxic unit (TU) at both locations.

**Fig 3:**
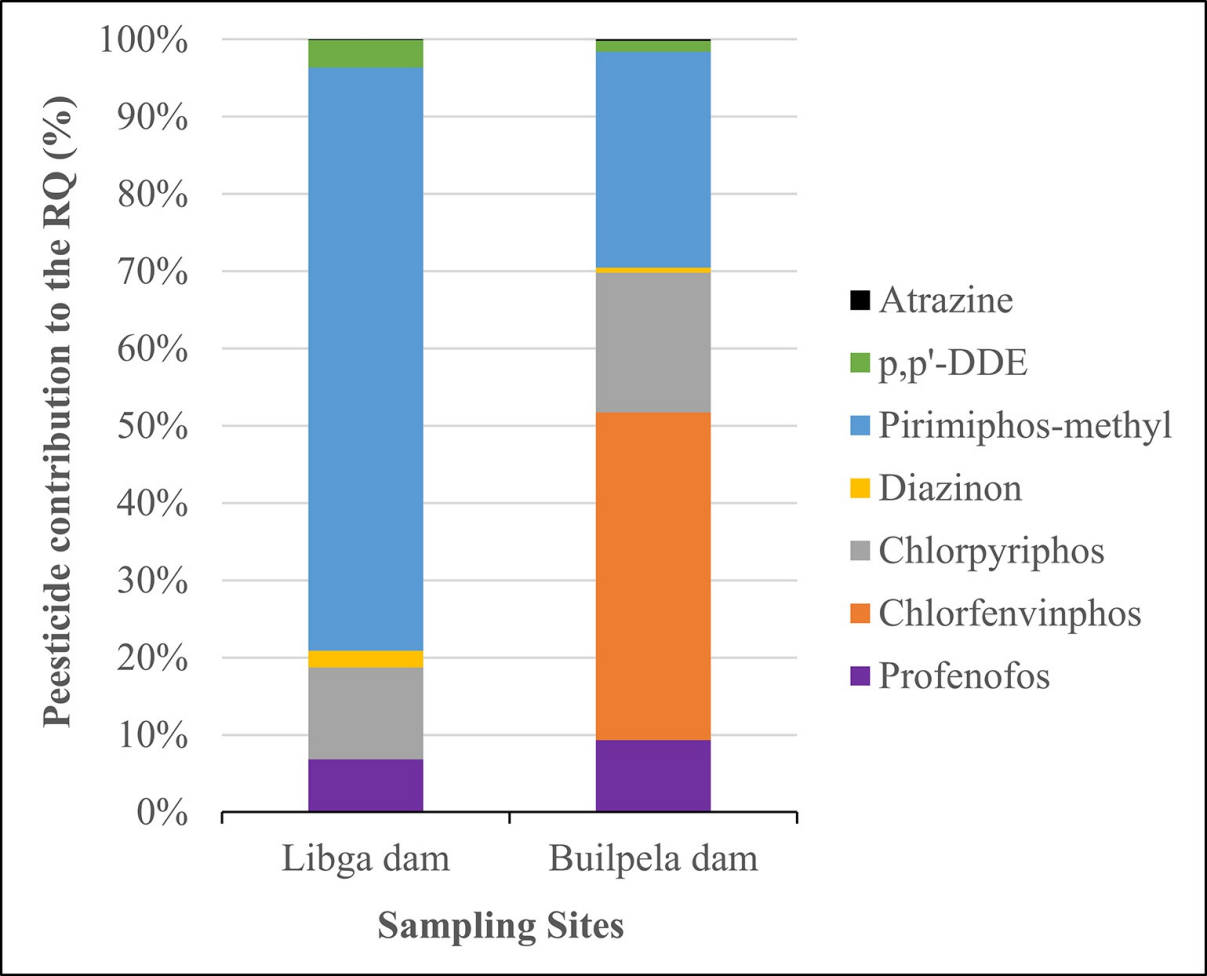
Pesticide contributions to risk quotients in surface water from Libga and Builpela dams in northern Ghana.

The contribution of individual pesticides to the risk quotients (∑RQ) in water samples from Libga and Builpela dams is shown in Fig 3. At Libga dam, pyrimiphos-methyl was the dominant contributor to ∑RQ, accounting for 75.49% of the risk, followed by chlorpyriphos (11.88%) and profenofos (6.84%). Other pesticides, such as p,p’-DDE, Diazinon, and Atrazine, contributed minimally, with percentages of 3.56%, 2.16%, and 0.07%, respectively. In contrast, Builpela dam exhibited a different profile, with chlorfenvinphos contributing the highest percentage (42.42%) to the ∑RQ, followed by pyrimiphos-methyl at 27.97% and chlorpyriphos at 18.09%. Profenofos also contributed significantly to Builpela dam ∑RQ (9.32%), whereas the contributions from p,p’-DDE, Diazinon, and Atrazine remained relatively low at 1.40%, 0.62%, and 0.18%, respectively. These findings indicate that while pirimiphos-methyl poses the greatest risk to the Libga dam, chlorfenvinphos is the most significant risk factor for the Builpela dam.

## Discussion

### Pesticide occurrence

#### Pesticide occurrence in surface water

In this study, chlorpyriphos was found in the surface water. At Libga and Builpela sites, the concentrations were significantly higher than the EU guideline limit of 0.1 μg/L. Chlorpyrifos is commonly used worldwide. The recorded quantities of this compound vary significantly depending on the type of activity conducted in the area and the sample season, with concentrations often being higher during the rainy season [35, 36]. However, Affum et al. [37] reported a lower concentration (0.340μg/kg) of chlorpyrifos from water samples in the Ankobra Basin (Ghana). This difference could be due to intensive land use related to cocoa production activities, to the detriment of extensive crops, and to the season of production. A lower concentration of chlorpyrifos was also found in surface water samples from the River Tano (0.158μg/L) [38], the Lake Bosomtwe (0.16μg/L) [39]. Profenofos and chlorfenvinphos were the second most prevalent pesticides that showed concerning levels, particularly in the Builpela dam. Profenofos was detected in all samples from the Builpela dam and in one-third of the samples from the Libga dam, with concentrations exceeding the EU limit. Few studies have reported concentrations of this compound in water (0.074μg/L and 0.303μg/L in dry and rainy seasons, respectively) [38]. Similarly, chlorfenvinphos, though not detected in the Libga dam, was present in 33% of the Builpela dam samples at levels substantially higher than the EU limits. Some studies have found this compound in water (0.2μg/L at Kanyarweru Sub County [40], 0.95μg/L in municipal effluent in Sydney [41]. The presence of profenofos and chlorfenvinphos residues may be attributed to poor disposal of pesticide containers, as some of the pesticide containers were disposed of near the water sources. However, profenofos and chlorfenvinphos may also have been released into the water via runoff after their application in intensive horticultural activities in the surrounding water bodies. Additionally, the study revealed that dizinon and pirimiphos-methyl were detected less frequently and at lower concentrations at the study sites. Studies have reported higher concentrations of dizinon and pirimiphos-methyl. For instance, Hasanuzzaman et al. [42] registered 16.58μg/L of diazinon in water samples from Nagarpur and Saturia Upazila in Bangladesh. Kuffour et al. [39], detected 0.28μg/L of Diazinon in water samples from Lake Bosomtwe in Ghana. Concerning Pirimiphos-methyl, Affum et al. [37] and Nyantakyi et al. [38] registered 0.162μg/L from the Ankobra Basin and 0.188μg/L from River Tano in Ghana. These concentrations are higher compared to the current study. The detection and concentration of these compounds could suggest lower usage or different environmental behaviours compared to other organophosphates detected in the study.

p,p’-DDE, an organochlorine pesticide, was found in 33% of the samples collected from the Libga dam and in 66% of the samples collected from the Builpela dam. Though the concentrations were below the EU limit, the presence of p,p’-DDE, a persistent organic pollutant, raises concerns about long-term environmental and health impacts. Additionally to the amount of p,p′-DDE found in the surface water of Kenya’s Lake Victoria Basin [43], the average quantity found in this study was higher than that found in drinking water and surface water samples from Lebanon’s Litani River and Lake Qaraoun (0.0011μg/L) [44], Tanzania’s Pangani River Basin (0.001μg/L) [45], China’s Xiaodian River Basin (0.0007μg/L), Hanjiang River Basin (0.0019μg/L) [46], and Ghana’s Ankobra Basin (0.020μg/L) [37]. The high values in our study could be attributed to a possible reload of this compound from the sediment or its possible illegal use.

Atrazine, a commonly used triazine herbicide, was detected in all samples collected from both sites. The mean atrazine concentration in Builpela dam significantly exceeded the EU limit, highlighting a severe contamination issue. The prevalence and significant presence of atrazine in the examined environmental matrix can be attributed to its status as a primary commercial pesticide (herbicide) in the pesticide market in northern Ghana [47]. However, the results of the present study were significantly higher than the values recorded in the studies by Pérez et al. [28] (0.04μg/L) in surface water from the Tapalque stream basin in Argentina. This pesticide has been banned or restricted in the European Union but it is currently used in Ghana as an agricultural pesticide or biocide.

#### Pesticide occurrence in sediment

Our findings indicated notable differences in contamination levels between the two sites, with Builpela dam generally exhibiting higher concentrations of most detected pesticides. The most common chemical found in the sediments from both sites was pirimiphos-methyl. The average concentration was much higher in the Libga dam (0.55mg/kg) than in the Builpela dam (0.05mg/kg). This contrast suggests potential differences in the application and environmental persistence of pirimiphos-methyl. Our results were higher than those reported by Nyantakyi et al. [38] (0.013mg/kg) during the dry season from the Tano River in Ghana. In contrast from the Tono dam in Navrongo of, Ghana, Akoto et al. [25] registered a concentration 2 times higher (0.104mg/kg) than our registered value in Builpela dam. However, it is 5 times lower than that recorded in the Libga dam in this study. The differences in contamination levels can be attributed to site-specific factors such as land-use patterns, proximity to farmlands, and hydrological conditions. Profenofos was found in samples from both sites but was higher in Builpela than in Libga. This is similar to what Akoto et al. [25] found, which was 0.050mg/kg from the Tono dam in Ghana. In the same country, Nyantakyi et al. [38] reported lower profenofos concentrations in the dry and rainy seasons (0.026mg/kg and 0.021mg/kg, respectively) from the Tano River. Chlorpyriphos is an insecticide that works on the nervous systems of fish, mammals, birds, and parasites by preventing the enzyme acetylcholinesterase from breaking down acetylcholine [39]. However, the use of this pesticide requires further investigation. It was detected less frequently, but at higher concentrations, specifically in the Libga dam. In River Tano, Ghana, Nyantakyi et al. [38] reported lower concentrations of chlorpyriphos in sediments during the dry and rainy seasons (0.017 and 0.021mg/kg, respectively). Lower concentrations of chlorpyrifos were also found in sediment samples from Argentina from the Paraguay-Paraná river (13.5μg/kg) [48], Medio stream (19μg/kg) [49], and Tapalque stream (4.2μg/kg) [28]. However, Mac Loughlin [50] reported a higher concentration of 272μg/kg chlorpyrifos in the Pampean Carnaval stream in Argentina. These variations may be attributed to the intensive land use for agricultural and horticultural practices to the detriment of extensive cultivation.

Research has demonstrated that organochlorine pesticides have a higher tendency to persist in aquatic species and significantly deposit on sediments [51, 52]. Our results indicated that organochlorine residues were present in varying amounts in sediments sampled from Libga and Builpela dams. Among the organochlorine pesticides, p,p’-DDD was consistently found in all samples from both sites, though it was more concentrated in Builpela dam (1.370mg/kg) than in Libga dam (0.844mg/kg). In the study by Akoto et al. [25], p,p’-DDD was registered at a concentration of 0.47mg/kg, which was almost 2 times lower than our results at both sites. Additionally, Buah-Kwofie & Humphries, [53] recorded a lower concentration of p,p’-DDD in sediment from Mfolozi and Mkhuze catchments (15ng/g and 14ng/kg, respectively) in Lake St Lucia, South Africa. Heptachlor and p,p’-DDE also showed high detection frequencies, with generally higher levels in Builpela dam. This suggests that these compounds may have been used in the past or that they were preserved better. The registered concentrations for p,p’-DDE from Builpela dam are similar to those found in studies by Akoto et al. [25] who found 0.70mg/kg in sediment from Tono dam in Ghana. However, this pattern is in contrast with the result in Libga dam, which is higher than the registered value by Akoto et al. [25]. It is important to notice that the presence of p,p’-DDE and p,p’-DDD in the sampling sites could be the result of DDT degradation into its more stable and persistent metabolites once released into the environment. The measured concentration in sediment from the sampling sites could be indicative of long-term degradation and widespread historical use of DDT in the region. Concerning heptachlor, the concentration in the sediment could be a reflection of the historical use in the study setting against termites and soil insects [53, 54]. Dieldrin was only detected in Builpela dam (0.71mg/kg in 33% of samples), suggesting localised contamination. Few studies have documented records of Dieldrin pesticide in sediment in Ghana. To our knowledge, only Danladi & Akoto, [55] registered this pesticide at a concentration (0.009mg/kg) lower than our study. Low concentrations (36ng/g and 21ng/g, respectively, for Mfolozi and Mkhuze catchments) of dieldrin, compared to our study, was also registered in sediment from Lake St Lucia in south Africa [53]. This indicates a lower incidence of dieldrin in agricultural practices in Ghana, and the actual concentration could be the result of long-term previous application.

Concerning atrazine, it was consistently detected in all of the sediment samples from both locations, with higher concentrations from Builpela dam (1.08mg/kg) compared to Libga dam (0.74mg/kg) in this study. However, none of these results are consistent with previous studies. In Argentina, low concentrations (1.00μg/kg) of atrazine were reported in the Tapalque stream basin [28] and (57μg/kg) in Pampean streams [29]. In Imoro et al. [47] study of pesticide availability, they argued that herbicides are one of the most pesticide sold in the northern region of Ghana. This could explain the high concentration of atrazine in our study settings. The higher levels of contamination of this pesticide in Builpela dam suggest more intense atrazine usage or greater runoff and accumulation. Additionally, the variations in sediment composition and water turnover rates between Libga and Builpela sites could influence the persistence and accumulation of these pesticides. Future studies should focus on identifying the specific sources and transport pathways of these pesticides. This may include detailed assessments of agricultural practices and runoff patterns. Monitoring over an extended period of time is necessary to identify patterns of pesticide contamination. Furthermore, conducting studies on the rates at which these pesticides degrade and interact in sediment ecosystems may provide valuable insights for management and risk assessment.

### Ecological risk assessment

#### Acute risk assessment

In this study, the acute risk (∑TU values) for sediment-dwelling organisms were higher in the Libga dam compared to Builpela dam. Both values exceed the threshold of 1. This indicates substantial ecological risk at both sites. The observed ∑TU values for sediment-dwelling organisms align with findings from a previous study on pesticide contamination in Argentina. For instance, studies by San Juan et al. [29] reported high toxic unit values for sediment-dwelling organisms in Pampean streams with intensive agricultural activities in five sampling sites. In all the study sites, no ecological acute risk was found for fish or algae in this study. However, an ecological acute risk for invertebrates was found for both sites (∑TU greater than 1). The risk was notably higher at Builpela dam compared to Libga dam. Invertebrate populations are critical for maintaining ecological balance in aquatic environments. These organisms play multifaceted roles in nutrient cycling, food web dynamics, habitat maintenance, and as indicators of ecosystem health. Their presence and activities support the structure and function of aquatic ecosystems. The higher ecological acute risk for invertebrates compared to fish and algae was not consistent with previous research indicating no acute risk for any of the taxa, including invertebrates [28]. This could be due to the low PNEC of invertebrate for the pesticide detected in this study. Due to their smaller size and distinct metabolic routes, invertebrates are frequently more susceptible to pesticide residues [56]. This may explain the greater ecological risk associated with their exposure.

#### Chromic risk assessment

The chronic toxicity risk assessment in surface water revealed significant ecological risks to aquatic biota at the Libga and Builpela dams. This aligns with studies by Olisah et al. [64] in South Africa, Pérez et al. [28], and San Juan et al. [29] in Argentina, which demonstrated the chronic impacts of organophosphates and other pesticides on aquatic life. These results suggest that the aquatic organisms (fish, invertebrate, and algae) present in water bodies can develop certain abnormalities or even death as a result of pesticide exposure. The ecotoxicological studies conducted on pesticides similar to those investigated in our study have established a correlation between exposure to pesticides and physiological stress [57]. Additionally, adverse effects on the central nervous system, morphological abnormalities, reduced shredding performance, and higher mortality have also been reported [58, 59]. Invertebrate could be more affected by the effects of these pesticides than fish and algae. This is probably due to the relatively low PNEC values of these organisms for the targeted pesticides. The Builpela dam showed a ∑RQ nearly four times higher than that of Libga dam. This disparity underscores a more severe ecological threat at Builpela dam. The increased risk at Builpela dam can be attributed to factors such as intensified agricultural practices and higher pesticide application rates, despite having less available agricultural land compared to the Libga dam. Additionally, differences in hydrological and sedimentary conditions can affect the persistence and bioavailability of pesticides. This further reveals the level of pollution along Builpela dam and a decline in ecological quality. Apart from the possible risk to aquatic species, the targeted pesticides are relatively hydrophobic with the potential to bioconcentrate and biomagnify across the food chain. This represents a major concern to human health [60].

#### Pesticide contribution to acute and chronic risks

With regards the pesticides’ contributions on the toxic unit (∑TU), our research showed that chlorpyriphos and chlorfenvinphos had the biggest proportion to the ∑TU values for organisms that lived in sediment at Libga and Builpela dams. The combined impact of the two pesticides constituted nearly 96% of the ∑TU at both dams. Previous research has shown that the insecticide chlorpyrifos is one of the main pesticides which affects the values of ∑TU for sediment-dwelling organisms [50]. Additionally, it has been assigned responsibility for the toxicological impacts of sediment organisms [50]. It has been argued that chlorfenvinphos exhibits a high level of toxicity against many aquatic invertebrates, such as crustaceans and insects [61]. Often, exposure may lead to high mortality rates within short time frames [62]. Sublethal concentrations can impair reproduction, behaviour [63] and growth [61] in invertebrates. This alarming contribution to the toxic unit of chlorfenvinphos needs effective regulation and management strategies to protect aquatic ecosystems from the adverse effects due to the pesticide.

Our study also revealed that in the acute risk for aquatic biota, chlorpyriphos emerged as the primary contributor to the ∑TU in invertebrate. This is similar to a previous study conducted in South African eutrophic estuaries, where Olisah et al. [64] reported chlorpyriphos having a high contribution and an acute risk to invertebrate (Daphnia) at all the sampling points. In our study, pirimiphos-methyl contributed 23.25% to the ∑TU at Libga dam, but its impact was substantially lower at Builpela dam (4.71%). Together, chlorpyriphos and pirimiphos-methyl accounted for almost 90% of the ∑TU at Libga dam, while chlorpyriphos, chlorfenvinphos, and pirimiphos-methyl collectively contributed 98.23% to the ∑TU at Builpela dam. Olisah et al. [64] reported the contribution of pirimiphos-methyl to ahigh mass loading of organophosphate pesticides in various regions of the eutrophic estuaries. The minimal contributions to the ∑TU in sediment and surface water at both locations of the other pesticides indicate their relatively lower impact on the overall acute ecological risk in sediment and surface water.

The identification of high-risk pesticides in non-target organisms and ecosystems represents a critical step in assessing and managing their potential impact. The assessment of pesticide contributions to the risk quotients (∑RQ) in water samples from Libga and Builpela dams showed clear differences in the profiles. Pirimiphos-methyl was the main factor contributing to the risk at Libga dam. This accounted for 75.49% of the total risk. On the other hand, chlorfenvinphos is the primary contributor at Builpela dam and represents about 42.42% of the total risk. This variation underscores the site-specific nature of pesticide contamination. Similar studies often report chlorpyriphos and atrazine as predominant risk contributors in various aquatic environments. This reflects different agricultural practices and pesticide usage patterns [28, 29]. In a study by Relyea and Hoverman [65], chlorpyriphos was a major risk factor in multiple freshwater ecosystems. However, this differs from the lower contribution found in our study. The differences observed could be attributed to regional agricultural practices, varying pesticide application rates, and local environmental conditions such as water flow and degradation rates. In a study by Olisah et al. [64], pirimiphos-methyl has also been reported to have a high risk and act as one of the major contributors to the chronic risk for invertebrate (Daphnia) in surface water from Sundays and Swartkops estuaries in South Africa.

## Limitation of the study

We utilized Toxic Units (TUs) and Risk Quotients (RQs) to assess the ecological risks of pesticide residues in selected dams in northern Ghana. While these methods are well-established for evaluating acute and chronic toxicity, we recognize the availability of more advanced approaches, such as Species Sensitivity Distribution (SSD), Hazard Quotients (HQs), and Landscape Ecological Risk (LER). Though TUs and RQs provided valuable insights, future studies should incorporate these advanced techniques to enhance the accuracy of ecological risk assessments. The detection methodology used, specifically the reliance on Gas Chromatography with an Electron Capture Detector (GC-ECD), which is primarily effective for organochlorine and some organophosphate pesticides should be viewed as a limitation of the study. This method may not detect more hydrophilic compounds, leading to potential underestimation in the Environmental Risk Assessment (ERA). Future studies should consider using advanced techniques, such as LC/MS-MS or QTOFs, to improve detection sensitivity and accuracy.

## Conclusion

This study analysed pesticide residues with a double matrix approach. Environmental concentration measurements were combined with an ecological risk assessment, using the RQ and TU approaches in northern Ghana. The main residues detected in surface water were chlorpyrifos, profenofos, and chlorfenvinphos. The main pesticide residues found in the sediment were pirimiphos-methyl, chlorpyrifos, atrazine, and organochlorines such as p,p’-DDE and p,p’-DDD. These chemicals show that pesticides have been used in the setting. The high acute risk at both dams was due to chlorpyrifos and chlorfenvinphos residues. Pirimiphos-methyl, chlorfenvinphos and chlorpyriphos were responsible for the high acute risk for invertebrates in surface water. A long-term risk was found in surface water at both sites. However, the RQ value was higher at Builpela dam due to large quantities of Pirimiphos-methyl and Chlorfenvinphos. The results of this study could be used to compare the pesticide pollution status of water bodies and other agricultural landscapes using standard species and classical endpoints for toxicity characterization. The results could also be used to guide effective regulation and management strategies to mitigate pesticide contamination in aquatic ecosystems.

## Supporting information

S1 Table Pesticides physicochemical and ecotoxicological characteristics used in TUs calculations in sediment. (PDF)

S2 Table Pesticides physicochemical and ecotoxicological characteristics used in TUs calculations in water. (PDF)

S3 Table Pesticides physicochemical and ecotoxicological characteristics used in RQs calculations in water. (PDF)
