# Supplementary material for: Occurrence of pesticide residues and associated ecological risks assessment in water and sediment from selected dams in northern Ghana
Source: PLoS One. 2024 Oct 21;19(10):e0312273. doi: 10.1371/journal.pone.0312273 (PMC11493270; doi:10.1371/journal.pone.0312273)
Supplement: S1 Table — (PDF) [file pone.0312273.s001.pdf]

S1 Table. Pesticides physicochemical and ecotoxicological characteristics used in TUs calculations in sediment

Compound	Pesticide type	Koc	Kd	BCF (l/kg)	CL50-96h (µg/L)	Species considered
**Profenofos**	Insecticide	2016	34.272	1186	500	*Daphnia magna*
**Chlorfenvinphos**	Insecticide	680	11.56	250	0.25	*Daphnia magna*
**Chlorpyriphos**	Insecticide	5509	126.6	1374	0.024	*Chironomus riparius*
**Pirimiphos-methyl**	Insecticide	1100	18.7	741	39	*Chironomus riparius*
**Aldrin**	Insecticide	17500	297.5	3348	28	*Daphnia magna*
**β-HCH**	Insecticide	1270	21.59	1300	40	*Chironomus riparius* (Lindane)
**Heptachlor**	Insecticide	24000	408	2430	42	*Daphnia magna*
**Dieldrin**	Insecticide	12000	204	35000	250	*Daphnia magna*
**p,p'-DDE**	Insecticide	-	50000	1800	-	-
**p,p′-DDD**	Insecticide	131000	2227	2.97E+05	9	*Daphnia magna*
**Atrazine**	Herbicide	100	1.7	4.3	1000	*Chironomus riparius*

Source: University of Hertfordshire Pesticide Properties DataBase (2024); United States Environmental Protection Agency ECOTOX database (2024)
