# Supplementary material for: Occurrence of pesticide residues and associated ecological risks assessment in water and sediment from selected dams in northern Ghana
Source: PLoS One. 2024 Oct 21;19(10):e0312273. doi: 10.1371/journal.pone.0312273 (PMC11493270; doi:10.1371/journal.pone.0312273)
Supplement: S2 Table — (PDF) [file pone.0312273.s002.pdf]

S1 Table. Pesticides physicochemical and ecotoxicological characteristics used in TUs calculations in water

Compound	Fish		Invertebrate		Algae	
	CL50 (µg/L)	Species	EC50 invertebrate (µg/L)	Species	EC50 Algae (µg/L)	Species
**Profenofos**	80	*Oncorhynchus mykiss*	500	*Daphnia magna*	n.a	-
**Chlorfenvinphos**	1100	*Oncorhynchus mykiss*	0.25	*Daphnia magna*	1360	*Scenedesmus subspicatus*
**Chlorpyriphos**	25	*Oncorhynchus mykiss*	0.1	*Daphnia magna*	480	Unknown species
**Diazinon**	3100	*Oncorhynchus mykiss*	1	*Daphnia magna*	6400	Unknown species
**Pirimiphos-methyl**	200	*Oncorhynchus mykiss*	0.21	*Daphnia magna*	1000	*Raphidocelis subcapitata*
**p,p'-DDE**	32	*Oncorhynchus mykiss*	1	*Bosmina longirostris*	n.a	-
**Atrazine**	4500	*Oncorhynchus mykiss*	85000	*Daphnia magna*	59	*Raphidocelis subcapitata*

n.a: not available

Source: University of Hertfordshire Pesticide Properties DataBase (2024)
