# Supplementary material for: Occurrence of pesticide residues and associated ecological risks assessment in water and sediment from selected dams in northern Ghana
Source: PLoS One. 2024 Oct 21;19(10):e0312273. doi: 10.1371/journal.pone.0312273 (PMC11493270; doi:10.1371/journal.pone.0312273)
Supplement: S3 Table — (PDF) [file pone.0312273.s003.pdf]

S3 Table. Pesticides physicochemical and ecotoxicological characteristics used in RQs calculations in water and PNEC calculated

Compounds	Fish		Invertebrate		Algae		AF	PNEC (ug/l)
	NOEC (µg/l)	Species Considered	NOEC (µg/l)	Species Considered	NOEC (µg/l)	Species Considered		
**Profenofos**	2	*Pimephales promelas*	n.a	n.a	n.a	n.a	100	0.02
**Chlorfenvinphos**	30	Unknown species	0.1	*Daphnia magna*	1000	Unknown species	10	0.01
**Chlorpyriphos**	0.14	*Oncorhynchus mykiss*	4.6	*Daphnia magna*	43	Unknown species	10	0.014
**Diazinon**	700	*Oncorhynchus mykiss*	0.56	*Daphnia magna*	10000	Unknown species	10	0.056
**Pirimiphos-methyl**	23	*Oncorhynchus mykiss*	0.08	*Daphnia magna*	n.a	n.a	50	0.0016
**p,p'-DDE**	**32***	n.a	n.a	n.a	n.a	n.a	1000	0.032
**Atrazine**	2000	*Oncorhynchus mykiss*	250	*Daphnia magna*	100	Green algae	10	10

* CL50 lindane was considered

n.a: not available

Source: University of Hertfordshire Pesticide Properties DataBase (2024)
